# N-Glycosylation of Antibodies: Biological Effects During Infections and Therapeutic Applications

**DOI:** 10.3390/antib14040093

**Published:** 2025-10-28

**Authors:** Jessica Castañeda-Casimiro, Luis Vallejo-Castillo, Eliud S. Peregrino, Alejandro Hernández-Solis, Luis Vázquez-Flores, Rommel Chacón-Salinas, Isabel Wong-Baeza, Jeanet Serafín-López

**Affiliations:** 1 Departamento de Inmunología, Escuela Nacional de Ciencias Biológicas (ENCB), Instituto Politécnico Nacional (IPN), Mexico City 11340, Mexico; jcastanedac@ipn.mx (J.C.-C.); astamaria@ipn.mx (E.S.P.); rchacons@ipn.mx (R.C.-S.); 2 Unidad de Desarrollo e Investigación en Bioterapéuticos (UDIBI), Escuela Nacional de Ciencias Biológicas (ENCB), Instituto Politécnico Nacional (IPN), Mexico City 11340, Mexico; lavallejos@ipn.mx; 3 Laboratorio Nacional para Servicios Especializados de Investigación, Desarrollo e Innovación (I+D+i) para Farmoquímicos y Biotecnológicos, LANSEIDI-FarBiotec-CONAHCYT, Mexico City 11340, Mexico; 4 Facultad de Medicina, Universidad Nacional Autónoma de México (UNAM), Mexico City 04360, Mexico; drhernandezsolis@yahoo.com.mx; 5 Servicio de Neumología del Hospital General de México “Dr. Eduardo Liceaga”, Secretaría de Salud, Mexico City 06720, Mexico; 6 Departamento de Bioquímica, Escuela Nacional de Ciencias Biológicas (ENCB), Instituto Politécnico Nacional (IPN), Mexico City 11340, Mexico; lvazquezf@ipn.mx; 7 Red de Salud del Instituto Politécnico Nacional, Mexico City 11340, Mexico

**Keywords:** antibodies, N-glycosilation, Fc fragment, infections

## Abstract

Antibodies are produced by cells of the adaptive immune response and recognize epitopes of microbial structures with high affinity and specificity. Antibodies are recognized by Fc fragment receptors (FcRs) found on the surface of phagocytic cells (neutrophils, monocytes, macrophages) and NK cells, among others. Hence, antibodies link the adaptive immune response with the innate immune response. The functions of antibodies are related to the N-glycosylation profile of these proteins. In this review, we describe how N-glycosylation of the Fc fragment of the different antibody classes is carried out, and which oligosaccharides are most commonly found in these antibodies. Subsequently, we summarize the biological effects of N-glycosylation of antibodies: on the binding of antibodies to FcRs (which affects various functions, such as antibody-dependent cellular cytotoxicity, antibody-dependent phagocytosis, and the production of pro- or anti-inflammatory chemokines and cytokines), on the ability of antibodies to activate complement and on the ability of some antibodies to directly neutralize the adhesion of bacteria and viruses to host cells (independently of Fab recognition). We describe how the N-glycosylation profile of antibodies is modified during certain infections (such as tuberculosis, COVID-19, influenza and dengue) and in response to vaccination, and the potential use of this profile to identify the stage and severity of an infection. Finally, we review the importance of N-glycosylation for the pharmacokinetic, pharmacodynamic and safety profiles of therapeutic monoclonal antibodies.

## 1. Introduction

Antibodies, also known as immunoglobulins, are glycoproteins that play a very important role in the immune response against pathogens. Antibodies make up 20% of the total proteins in plasma; therefore, they are one of the most abundant proteins in human blood. In humans, five isotypes have been identified: IgG (70–85% of total antibodies), IgM (5–10%), IgA (5–15%), IgE (<0.25%) and IgD (~0.25%) [1]. All human antibodies have a similar composition, 82–96% protein and 4–18% carbohydrate (unless otherwise indicated, all the antibodies mentioned in this review are human). Each antibody monomer is made up of two equal heavy chains and two equal light chains, and each of these monomers has a crystallizable fragment (Fc) and two antigen-binding domains (Fab) that form the variable regions of the antibody. In addition, antibodies differ in their size, charge, amino acid sequence, and effector functions, as well as in their glycosylation sites [2].

Antibodies link the innate and adaptive immune response, as they can bind simultaneously to an antigen by the Fab region and to an innate effector cell by the Fc region, through Fc receptors (FcRs) found on their surface. The FcRs that recognize IgA, IgE, IgM and IgD are FcαR, FcεR, FcμR and FcδR, respectively, while the FcRs that recognize IgG are FcγR and FcRn. There are high-affinity and low-affinity FcγRs, and both types of FcγRs can bind antigen-IgG immune complexes or IgG aggregates with high avidity [3]. In addition, there are activating (FcγRI, FcγRIIa, FcγRIIc, FcγRIIIa, and FcγRIIIb) and inhibitory (FcγRIIb) isoforms of FcγRs [4]. When effector cells of the immune response recognize antibodies through FcRs, they are activated to perform different functions, such as antibody-dependent cellular cytotoxicity (ADCC), activation of the complement cascade, antibody-dependent phagocytosis, and the production of cytokines, chemokines and/or enzymes [5].

The most abundant antibody is IgG, and it has four subclasses that were named in descending order of their amount: IgG1 (60–70% of total IgG), IgG2 (20–30%), IgG3 (5–8%) and the least frequent IgG4 (5%). The IgG subclasses exhibit more than 90% homology in the amino acid sequence of their constant domains but differ in the hinge region and the CH2 domains [1]. In addition, each IgG subclass has a different profile with respect to antigen binding, immune complex formation, complement activation, effector cell activation, placental transport, and half-life. The half-life of IgG1, IgG2 and IgG4 is 22 days, while the half-life of IgG3 is seven days if it does not bind to its receptor [6]. IgG1 and IgG3 induce several of the functions mediated by FcγRs and by complement. IgG2 participates in the response against polysaccharides and is a weak inducer of FcγR- and complement-mediated functions [6]. IgG4 has a minimal capacity to activate effector cells and complement, and this low activation capacity prevents excessive inflammation in allergies and infections, induces tolerance and prevents tissue damage [7].

IgA has two subclasses, IgA1 and IgA2. In plasma, it is found in its monomeric form and in mucosal surfaces it is found in dimeric form. It is essential for controlling pathogenic microorganisms on mucosal surfaces, such as in the gastrointestinal, genitourinary and respiratory tracts. Additionally, it is important in mediating anti-inflammatory or pro-inflammatory responses by binding to monocyte and neutrophil FcαRI, in soluble or dimeric form, respectively [8].

IgM is predominantly found in its pentameric form, but it can also be present in small amounts as hexamers or monomers. The IgM heavy chains are made up of 5 domains (VH, Cμ1, Cμ2, Cμ3 and Cμ4) and the light chains are made up of the VL and CL domains. IgM plays important roles during the immune response, such as promoting affinity maturation, maintaining mucosal (gut and lung) homeostasis, promoting mucosal tolerance, and regulating microbiota conformation along with IgA [9]. In addition, they are better complement activators than IgGs [10].

IgD is part of the B-cell receptor, so it is inserted in the membrane of mature B cells, and it can be released to the serum as part of the primary response of activated B cells [11]. IgD has low serum concentrations and a relatively short half-life (2.8 days). It can recognize antigens and accelerate the maturation of the T cell-dependent primary response, but it can also interfere in IgE-mediated basophil degranulation, suggesting a role in the development of allergen tolerance [12]. 

IgE is an important antibody for the development of allergic disorders. IgE binds to the surface of basophils and mast cells that express FcεRI, and when IgE recognizes an allergen, signaling is triggered that leads to degranulation of these cells and to the release of mediators such as histamine, prostaglandins, and leukotrienes, which are responsible for the characteristic allergy symptoms [13]. The second IgE receptor is FcεRII (CD23), which has a lower affinity for IgE than FcεRI, and has been associated with a non-inflammatory activation pathway. This receptor regulates plasma IgE levels and plays a role in antigen presentation [14].

## 2. N-Glycosylation of the Fc Fragment of Antibodies

The post-translational modifications of antibodies include glycosylation, amino acid modifications and oxidations [15]. Glycosylation is a process carried out by enzymes on more than 85% of the secreted proteins and is a process that is conserved in prokaryotes and eukaryotes. Glycosylation can occur in two forms: O-glycosylation (oligosaccharides are attached to the oxygen atom of a serine or threonine hydroxyl group) and N-glycosylation (oligosaccharides are attached to the nitrogen atom of an asparagine side chain amino group). In O-glycosylation, N-acetylglucosamine (GlcNAc) is attached to the amino acids serine or threonine of nuclear and cytoplasmic proteins. This type of glycosylation is dependent on two enzymes, O-GlcNAc transferase (OGT) and O-GlcNAcase (OGA) [16]. N-glycosylation is a post-translational modification in which a covalent bond is formed between asparagine residues of peptide chains and oligosaccharides. Subsequently, the central core of the structure is expanded by repeated elongation/branching steps, resulting in structural motifs [17]. 

In mammals, glycosylation is a complex process that involves several reactions catalyzed by glycosyltransferases. In the first step, glycosyltransferases transfer carbohydrates from high-energy intermediates, such as lipid-linked sugars or activated sugar nucleotides, to the acceptor protein [18]. Most of the glycosyltransferases of the endoplasmic reticulum (ER) and the Golgi apparatus use nucleotide sugars [CMP-N-acetylneuraminic acid (Neu5Ac, the predominant type of sialic acid in humans), GDP-mannose (Man)/fucose (Fuc), UDP-galactose (Gal) and UDP-GlcNAc] as precursors for the glycosylation reactions, while some ER glycosyltransferases use dolichol phosphate (Dol-P) as the sugar donor [17,19].

**Figure 1:**
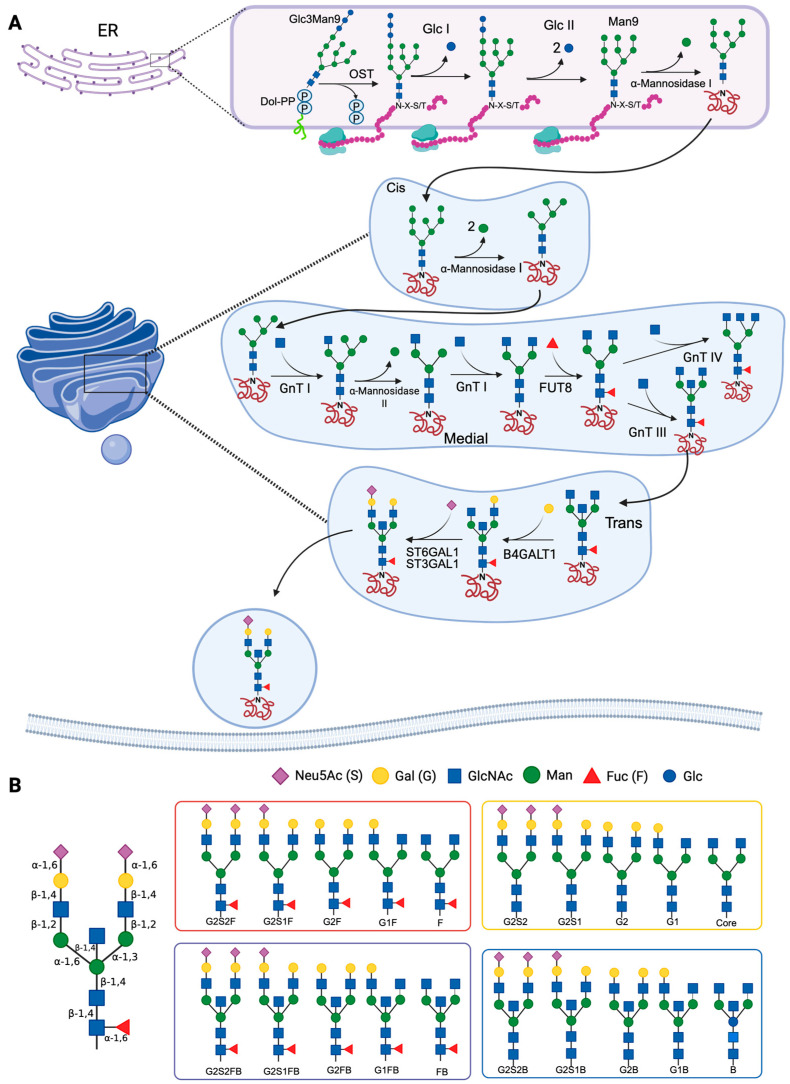
Mechanisms of N-glycosylation and main antibody N-glycans. (**A**) The synthesis of N-glycans begins in the endoplasmic reticulum (ER), where the dolichol diphosphate (Dol-PP)-linked precursor Glc3Man9GlcNAc2 is synthesized. An oligosaccharyl-transferase (OST) catalyzes the transfer of the oligosaccharide to an asparagine residue of a protein (in the Asn-X-Ser/Thr, or N-X-S/T motif). In the Golgi apparatus, Man is removed by the action of α-mannosidases I and II, and subsequently, N-glycans are modified by the addition of carbohydrates. Several glycosyl-transferases are involved in this step (N-acetylglucosaminyl-transferases [GnT] I-IV, α-2,3-sialyl-transferase 1 [ST3GAL1], α-2,6-sialyltransferase 1 [ST6GAL1], β-1,4-galactosyl-transferase 1 [B4GALT1], and α-1,6-fucosyl-transferase [FUT8]). (**B**) Schematic of the main N-glycans that are present in antibodies: G, galactosylated; F, fucosylated; B: bisecting or bifurcated; S, sialylated. References: Reily et al., 2019 [23]; Stanley et al., 2022 [24,25]. Created in https://BioRender.com (accessed on 19 August 2025).

In the cytosolic side of the ER, the first reaction of the glycosylation process involves the formation of a lipid carrier consisting of a branched oligosaccharide of GlcNAc and Man linked to Dol-P. Subsequently, the lipid precursor is translocated into the ER lumen, where Man and glucose (Glc) units are transferred to form a Glc3Man9GlcNAc2 polymer. An oligosaccharyl-transferase (OST) catalyzes the transfer of the oligosaccharide to a protein, specifically to an asparagine residue found in the sequence Asn-X-Ser/Thr (X can be any amino acid other than proline), forming a carbohydrate–protein complex, from which Glc units are removed as part of the processing in the ER [20]. In the next steps of the synthesis, the carbohydrate–protein complex is processed by the action of mannosidases, such as α-1,2-mannosidase 1 in the ER and α-1,2-mannosidases IA, IB and IC in the Golgi apparatus, which remove Man residues to form Man5GlcNAc2. Subsequently, in the intermediate region of the Golgi apparatus, GlcNAc-transferase I adds a GlcNAc residue, and mannosidase II removes two terminal Man residues from the hybrid N-glycan, forming GlcNAcMan3GlcNAc2 [21,22]. Finally, in the intermediate and trans regions of the Golgi apparatus, GlcNAc, Gal, sialic acid and/or Fuc are added (Figure 1A) [23,24]. The synthesis process is dependent on the physiological conditions of the cell; therefore, the glycosidases and glycosyltransferases that participate in the reactions described above are expressed differentially, and specifically, depending on the protein, cell type, and species [24].

The processing of the oligosaccharide Glc3Man9GlcNAc2 is carried out in the ER and the Golgi apparatus. In most glycoproteins, mannosidases and glycosyltransferases of the Golgi apparatus perform various elongation and branching reactions, resulting in complex N-glycans (Figure 1B). The branches are lengthened by the addition of Gal by the action of galactosyl-transferases, and end with sialic acid residues transferred by sialyl-transferases [26].

N-glycosylation of IgG Fc is carried out in antibody-secreting cells (plasmablasts and plasma cells). The enzyme β-1-4-N-acetylglucosaminyl-transferase (GnT-III) adds a bisecting GlcNAc, and the α-1,6-fucosyl-transferase (FUT8) adds Fuc with α-1-3 or α-1-6 linkages. The presence of FUT8 has been shown to inhibit the addition of the bisecting GlcNAc. The enzyme β-1,4-galactosyltransferase 1 (B4GALT1) adds Gal to one or both arms of the oligosaccharide, and if the glycan has Gal, sialic acid can be added. Sialylation is catalyzed by α-2,6-sialyltransferase 1 (ST6GAL1), which preferentially adds sialic acid on the α-1,3 arm of the biantennary glycan (Figure 1A) [20,27,28]. Glycans at position Asn297, which is usually the only N-glycosylation site of the IgG Fc, can establish the quaternary structure of the Fc and modify the stability of the IgG. Glycans attached to this position may also regulate certain effector functions, such as ADCC and complement-dependent cytotoxicity [1]. The Asn297 glycosylation site is conserved in all IgG subclasses, but IgG3 has an additional site in Asn392, which is glycosylated in only 12% of IgG3. The Asn392 glycosylation site is characterized by the presence of biantennary glycans without a central Fuc, and it can have a bisecting GlcNAc. Around 9% of the IgG3 that are glycosylated in Asn392 have glycans formed by 5 Man residues [29].

**Figure 2:**
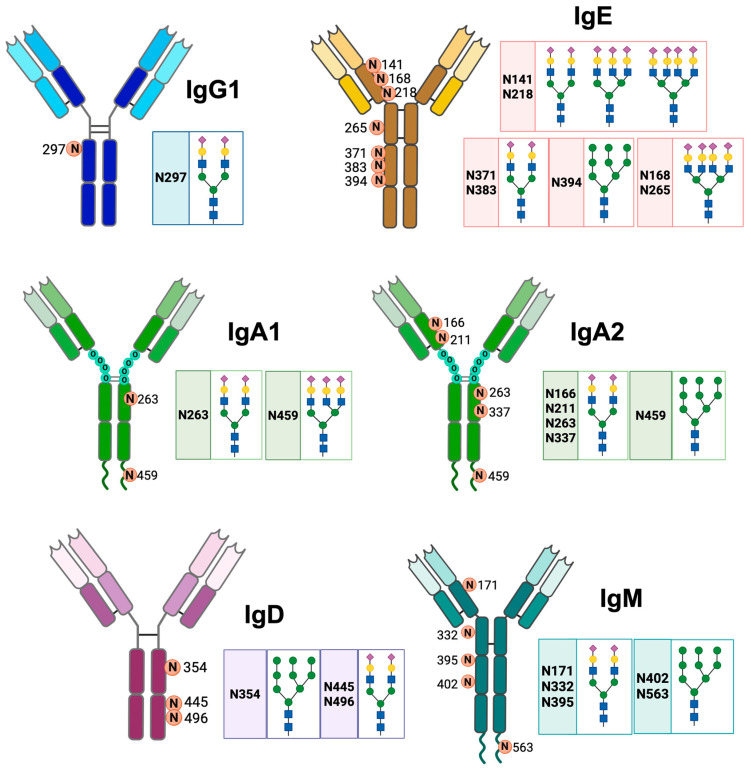
N-glycosylation sites of the different classes of human antibodies. The figure shows the general structure of IgG1, IgA1, IgA2, IgM, IgD, and IgE antibodies, the asparagine residues (N) where N-glycosylation occurs, and the structure of their glycans. Neu5Ac (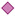), Gal (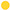), Man (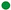), GlcNAc (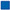). Created in https://BioRender.com (accessed on 19 August 2025).

IgA has different N-glycosylation sites. The two IgA isotypes have two N-glycosylation sites at Asn263 of the Cα2 domain and Asn459 of the Fc [30]. The Asn263 glycan has a biantennary sialic acid complex with α2-6 bonds; this complex is located on the outside of the Fc and provides stability to monomeric IgA without modifying its interaction with FcαR1. The Asn459 glycan has a triantennary sialic acid complex with α2-6 linkages; this glycosylation site on IgA2 has complex glycans with Man, but approximately 40% of the antibodies do not exhibit glycosylation at this site [2,31]. Human IgA2 additionally has more glycosylation sites at Cα1 Asn166, Cα2 Asn337, and Cα1 Asn211 (Figure 2). These sites incorporate glycans that can neutralize viruses, in addition to inducing a pro-inflammatory response in macrophages and neutrophils [32].

IgM has five conserved N-glycosylation sites: Asn171 (Cμ1), Asn332 (Cμ2), Asn395 (Cμ3), Asn402 (Cμ3), and Asn563 (Fc tail), of which the Asn171, Asn332, and Asn395 sites are exposed (Figure 2) [2]. IgE is the most glycosylated antibody; approximately 12% of its weight corresponds to glycans. Human IgE has seven N-glycosylation sites, and murine IgE has nine sites. The glycosylation sites of human IgE are Asn141, Asn168, Asn218, Asn265, Asn371, Asn383, and Asn394 [14]. The Asn394 site has oligomannose glycans; Asn371 and Asn383 have biantennary complexes; Asn168 and Asn265 sites mostly have tetra-antennary complexes; and Asn141 and Asn218 sites have bi-, tri-, and tetra-antennary complexes. The complex glycans of IgE have Fuc attached to their backbones and Gal and sialic acid attached to the terminal structure (Figure 2) [33].

IgD has three N-glycosylation sites: Asn354, Asn445 and Asn496. As354 has exclusively oligomannose glycans (with 5 to 9 Man residues), while Asn445 and Asn496 have galactosylated, sialylated, fucosylated and bisected complex glycans. In Asn445, more than 40% of the oligosaccharides have a Fuc residue, with or without bisection [34]. In addition, 45% of serum IgD is sialylated in Asn445; of this sialylated IgD, 24% is mono-sialylated and 21% is di-sialylated [11]. In patients with multiple myeloma, the glycosylation profile of IgD is altered, with increased fucosylation and increased mono-galactosylation of the oligosaccharides. This glycosylation profile could be used for diagnostic purposes [35].

Little is yet known about the role of N-glycans in the humoral immune response. However, autoantibodies in rheumatoid arthritis, primary Sjögren’s syndrome, and some follicular lymphomas have been associated with high levels of variable domain glycosylation [36]. Glycosylation of proteins can create a large repertoire of glycoforms, and each of these can confer different functions on proteins. The effects of N-glycosylation on antibody function have been extensively studied in IgG and, to a lesser extent, in IgA, IgM, and IgE.

## 3. N-Glycosylation of the Fab Fragment of Antibodies

In IgGs, in addition to the N-glycosylation site at Fc Asn297, N-glycosylation sites can be generated in the variable domains during somatic hypermutation. These N-glycosylation sites are predominantly acquired at variable domain positions where single-nucleotide mutations convert a latent N-glycosylation site into an actual N-glycosylation site [36,37]. Van de Bovenkamp et al. observed that the introduction of glycans into the Fab increases antibody stability, suggesting an in vivo selection of stable glycosylated antibodies [36]. N-glycosylation sites can be generated at positions that affect antigen binding and interfere with antibody affinity, especially if several N-glycosylation sites are clustered. It has been observed that there are different levels of Fab glycosylation between IgG subclasses, demonstrating that this type of glycosylation is not a random process and that it is associated with antigens. IgG4 has a higher level of glycosylation in the Fab region than the other IgGs, which may be due to its slightly higher mutation rate [37]. In addition to the structural properties provided by the glycans in the Fab, it has been reported that the presence of sialic acid in the Fab can block the binding of the influenza virus to its cellular receptors, since the sialic acid of the Fab mimics sialylated cellular receptors [38].

## 4. Biological Effects of N-Glycosylation of Antibodies

### 4.1. Effects of N-Glycosylation on Antibody Conformation

N-glycans stabilize the conformation of the secondary structure of proteins, since they modulate the folding of residues around the Asn-X-Ser/Thr sequence. The interaction of the N-glycan with the peptide chain occurs mainly in β turn structures. In addition, glycosylation has been shown to enhance protein stability and resistance to proteolysis, compared to non-glycosylated proteins [39].

IgG N-glycans contribute to the structural conformation of these antibodies. The presence of glycans at Asn297 confers an open conformation to the Fc fragment, and enzymatically deglycosylated IgG Fc fragments have a closed conformation. The addition of α2,6-linked sialic acid to the Fc fragment induces a tighter conformation compared to other glycans, suggesting an increase in the flexibility of the CH2 domain. In addition, sialylation changes Fc binding to FcγRs, which is associated with a shift from pro-inflammatory to anti-inflammatory profile [40].

Deglycosylated IgE can form aggregates, so it was suggested that glycans contribute to the solubility of IgE [41]. The absence of the oligomannose complex at position Asn394 modifies the secondary structure of IgE, indicating that this oligosaccharide is important for proper IgE folding [33].

### 4.2. Effects of N-Glycosylation on Antibody Binding to Fc Receptors

**Figure 3:**
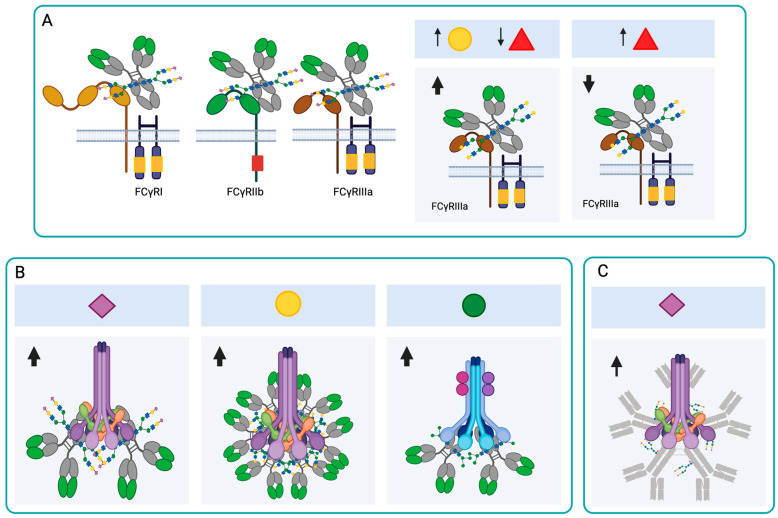
Effect of N-glycosylation on IgG and IgM antibody functions. (**A**) The presence of the N-glycans at Asn297 is important for the interaction of IgGs with FcRs. The increase in galactosylation and the reduction in fucosylation increase the affinity of IgG1 for FcγRIII, while the presence of Fuc interferes with the binding of IgG1 to this receptor. (**B**) Sialylation increases the binding of IgGs to C1q and induces complement activation through the classical pathway. The presence of Gal induces IgG hexamerization and complement activation through the classical pathway. The presence of Man in IgGs can activate complement through the lectin pathway. (**C**) In IgM, sialic acid-rich N-glycans promote complement activation through the classical pathway. Neu5Ac (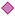), Gal (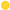), Man (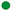), Fuc (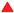), increase (↑), decrease (↓). Created in https://BioRender.com (accessed on 19 August 2025).

FcγRs have an Fc fragment-binding domain in their extracellular portion and an intracellular tyrosine activator or inhibitor (ITAM or ITIM) domain. The receptors with the activation domain are FcγRI (CD64), FcγRIIa (CD32), FcγRIIc, and FcγRIIIa/b (CD16), and the receptor with the inhibition domain is FcγRIIb [42]. The affinity of the Fc fragment to the different FcγRs is determined by differences in the amino acid sequence of the four IgG subclasses and by the structure and composition of the Fc-associated glycan (Figure 3A). These glycoforms diversify the Fc domain, resulting in Fc domains with different abilities to interact with and activate leukocytes expressing FcγRs [43]. Kao et al. (2015) demonstrated that IgG1 and IgG3, which have monosaccharides or disaccharides with GlcNAc at the N-glycosylation site (with or without Fuc residue) instead of the full oligosaccharide, have less ability to activate complement [44]. This study demonstrates that a mono- or disaccharide structure, consisting of GlcNAc with or without a branched Fuc residue, is sufficient to retain the activity of the most active IgG subclasses in vivo, and further directs antibody activity to cellular Fc Rs. 

FcγRIIIa is found primarily on NK cells, macrophages, and monocytes. Although it has a medium affinity (KD ≈ 400 nM) for the Fc fragment, the strength with which it binds to IgG1 and IgG3 correlates with ADCC and its therapeutic effects. FcγRIII has five N-glycosylation sites, and the Asn162 site is related to the glycan interaction with the IgG Asn297 site. Removing the Man present at the Asn162 site of FcγRIIIa induces an increase in its affinity for IgG1 [45]. Glycans at the Asn162 site of FcγRIII regulate its affinity for antibodies, because they reduce the interaction space and stabilize the receptor by intramolecular protein-carbohydrate interactions [46].

The presence of Fuc at the Asn297 site interferes with the binding of IgG1 to FcγRIII (CD16), and afucosylated IgG1 has a 40-fold greater affinity for FcγRIII if its galactosylation is also increased (Figure 3A) [47,48]. Usually, afucosylated IgG1s are scarce in circulation, since Fc fucosylation levels are greater than 90%. However, high levels of afucosylated IgG1 have been observed in individuals with malaria, during infections with human immunodeficiency virus (HIV), dengue and SARS-CoV-2, and in antibodies against alloantigens in blood cells [49,50,51,52]. Additionally, the combination of lower fucosylation with a higher level of galactosylation increases the affinity of IgG FcγRIII (Figure 3A) [53].

In healthy individuals, 10 to 15% of plasma IgG is sialylated, and the most abundant glycoform is mono-sialylated. The α2,6-terminal sialic acid of the Fc fragment has an anti-inflammatory function, and its presence on IgG results in a lower affinity for activating FcγRs [54,55] and a higher affinity for type II FcRs, such as DC-SING and CD23 [56]. The glycosylation of the Fc fragment of IgG does not affect its binding to FcγRs or FcRn, but Fc digalactosylation increased its binding affinity to FcRn [57].

**Figure 4:**
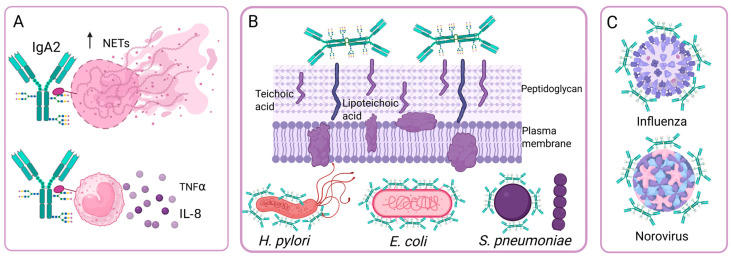
Effect of N-glycosylation on IgA antibody functions. (**A**) IgA2 is poorly sialylated and has pro-inflammatory effects. (**B**) The oligosaccharides of sIgA bind to bacterial adhesins, so sIgA inhibits bacterial adhesion independently of Fab recognition. (**C**) IgA neutralizes viruses independently of Fab recognition through the binding of sialic acid receptors to the sialic acid present on sIgA. Created in https://BioRender.com (accessed on 19 August 2025).

IgA bound to the surface of microorganisms can bind to FcαRI (CD89), which is found on neutrophils, macrophages, and monocytes, and this binding induces an inflammatory response, activating phagocytosis and superoxide dismutase production, cytokine release, and ADCC. In contrast, the binding of IgA without antigen to FcαRI has an inhibitory effect on the ITAM domains, inducing an anti-inflammatory response by inhibiting respiratory burst, antibody-dependent phagocytosis, and cytokine production [58]. Studies have been conducted to determine whether the glycosylation profile of IgA modifies its interaction with FcαRI, as is the case with IgGs and their receptors. One study showed that the binding of IgA to its receptor does not depend on its N-glycans [59]. However, another study [32] showed that IgA2, which is poorly sialylated, has pro-inflammatory effects since it induces the formation of NETs and the production of IL-8 and TNFα through FcαRI (Figure 4A). In contrast, IgA1 exhibits greater sialylation and induces fewer NETs, and sialic acid removal of IgA1 N-glycans increases its pro-inflammatory capacity to levels comparable to those of IgA2 [32]. These results suggest that, in some cases, N-glycans can modify the binding of IgA to FcαRI. In contrast, the affinity of the IgA-FcαRI interaction is significantly regulated by the N-glycans of FcαRI [60].

In the case of IgM, glycosylation at the Asn402 site is important for the binding of these antibodies to their cellular receptors, as is glycosylation at the Asn297 site of IgGs. Asialylated IgM remains bound to its receptors, while sialylated IgM is internalized [2]. In T cells, internalization of sialylated IgM induces suppression of the response of these cells. When sialic acid residues are removed from IgM, its inhibitory functions are removed, demonstrating the important role of sialylated N-glycans in IgM-mediated suppression [61]. Finally, in the case of IgE, the oligomannose at site Asn394 is important for the interaction of IgE with FcεRI on mast cells to initiate inflammation in response to an allergen [33]. The IgE of allergic people has higher levels of sialylation than the IgE of non-allergic people. Removal of sialic acid from IgE does not modify the allergen-IgE-FcεRI interaction, but it does attenuate effector cell degranulation and anaphylaxis in several models of allergy, and these effects are related to a decrease in Syk phosphorylation [13].

### 4.3. Effects of N-Glycosylation on the Ability of Antibodies to Activate Complement

In IgG, the terminal Gal of the N-glycan of the Asn297 site increases the binding of the antibody to C1q and thus activates the classical complement pathway [42]. This increase is induced because the terminal Gal changes the three-dimensional conformation of the Fc and thus increases the hydrophobicity in the CH2 domain. The change in conformation facilitates the hexamerization of IgG1s, thereby increasing their avidity for C1q (Figure 3B) [62]. Dekers et al. demonstrated that elevated galactosylation and sialylation significantly increase the binding of IgGs to C1q, independently of fucosylation (Figure 3B). Thus, fucosylation and galactosylation are primary modulators for FcγRs- and complement-mediated IgG effector functions, respectively [48]. The lectin pathway can also be activated by N-glycans with Man present on IgGs (Figure 3B). Agalactosylated N-glycans bind to Man-binding protein (MBP) and induce complement activation through this pathway, which may induce a chronic inflammatory profile in people who have agalactosylated antibodies [63].

In the case of IgMs, N-glycans rich in Man and sialic acid are known to promote complement activation (Figure 3C) [2]. In COVID-19 patients, asialylated IgM was found to reduce complement activation [10]. In the case of IgA, the secreted IgA (sIgA) heavy chains A1 and A2 have terminal GlcNAc and Man residues that are masked by the secretory component. These glycans can be exposed by disrupting the non-covalent interactions of the heavy chain with the secretory component and can then be recognized by MBP [64].

### 4.4. Effects of IgA N-Glycosylation on Its Ability to Neutralize the Adhesion of Bacteria and Viruses

IgAs have different glycosylation patterns according to their origin: sIgA in breast milk and saliva has 70 different N-glycan structures, while plasma IgA has 55 structures. Breast milk sIgA has higher levels of fucosylation, galactosylation and sialylation and reduced levels of bisecting GlcNAc, compared to colostrum sIgA, and these changes could indicate that maternal IgAs modify their N-glycosylation to protect neonates from infectious diseases [65].

During bacterial infections, an important function of glycans is to decrease bacterial adhesion to host cells. In sIgA, heavy chain O-glycans and secretory component N-glycans contain oligosaccharides that can bind to bacterial adhesins, such as Galβ1-4/3GlcNAc, Fucα1-3/4GlcNAc, Fucα1-2Gal, and sialic acid with α2-3/6 bonds. These oligosaccharides provide sIgA with additional bacterial binding sites, in addition to the four specific epitope recognition sites on the Fab. These oligosaccharides effectively prevent bacterial adhesion to epithelial cells, so sIgA participates in both innate and adaptive immunity. This effect has been observed on *Helicobacter pylori*, *Escherichia coli*, *Clostridium difficile* and *Streptococcus pneumoniae* (Figure 4B) [64]. Inhibition of *E. coli* adhesion occurs in strains with S fimbriae, and inhibition occurs by specific interactions between sialyl-oligosaccharides of sIgA and bacterial adhesins [66]. Goonatilleke et al. demonstrated that breast milk sIgA N-glycans bind to lectins and adhesins from enteropathogenic *E. coli*, *Campylobacter jejuni*, and *H. pylori*, and from Norovirus (Figure 4C) [65]. In addition, the N-glycans of sIgA can bind to the peptidoglycan and teichoic or lipoteichoic acids of the cell wall of Gram-positive bacteria to prevent their adhesion. Removing these N-glycans reduces sIgA binding to Gram-positive bacteria [67].

The neutralizing capacity of IgA against influenza A virus (H1N1 and H5N3) is greater than that of IgG and is associated with the presence of sialic acid with α2,3-linkages at the C-terminus of IgA (Figure 4C). This neutralizing ability is due to sialic acid specifically binding to virus neuraminidases and interfering with virus binding to its cellular receptor, and it is independent of Fab recognition. It is also observed in other enveloped viruses whose receptors contain sialic acid, such as the Newcastle virus [68]. 

## 5. Modification of Antibody N-Glycosylation During Infections

### 5.1. Modification of IgM N-Glycosylation in SARS-CoV-2 Infection

During SARS-CoV-2 infection, the IgMs of patients with severe COVID-19 have reduced mannosylation, compared to the IgMs of patients with mild or moderate COVID-19. In addition, IgMs from severe patients contain fewer M4-M6 oligomannose structures and more M7-M10 oligomannose structures compared to non-severe patients, and this effect is mainly due to severe patients having decreased expression of mannosidases 1 and 2 (MAN2A1 and MAN1A2) [10]. Severe patients also exhibit increased expression of the sialyl-transferase STL3GAL4, so their total IgMs and their IgMs specific against the S1 protein have a higher α2-3sialic acid content; the presence of this glycosylation suggests that these antibodies exert pro-inflammatory effects. Finally, IgMs from severe patients were reported to be more efficient at activating complement, and this activity is attributed to the interaction of sialic acid and Man with C1q [10].

### 5.2. Modification of IgG N-Glycosylation in Tuberculosis

**Figure 5:**
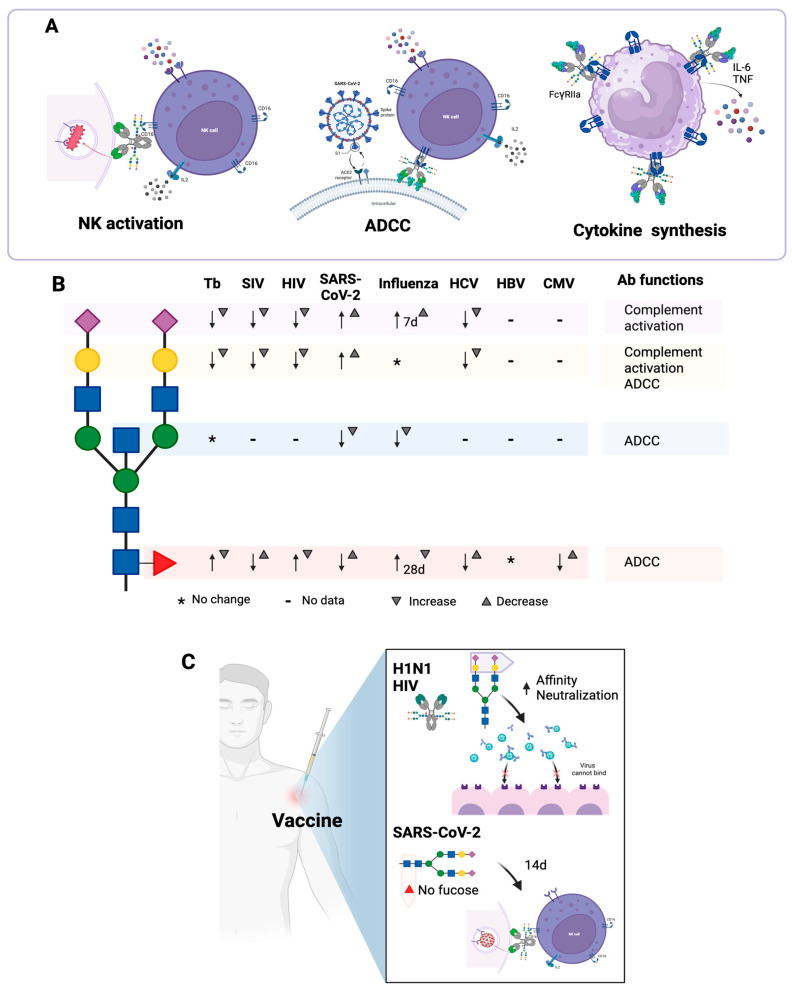
Effector functions of antibodies that are dependent on N-glycans and that are modified during infections. (**A**) IgGs from individuals with active tuberculosis exhibit greater agalactosylation and greater fucosylation than IgGs from individuals with latent tuberculosis, induce lower antibody-dependent cellular cytotoxicity (ADCC) and have less ability to activate IFNγ production by NK cells. During infection with SARS-CoV-2, IgG antibodies with low fucosylation activate NK cells through FcγRIIIb and activate macrophages through FcγRIIIa; in the latter case, TNFα and IL-6 production is induced. (**B**) Changes in the N-glycosylation profile of antibodies during some infections. In tuberculosis (Tb) and infections with certain viruses [simian immunodeficiency virus (SIV), human immunodeficiency virus (HIV), SARS-CoV-2, influenza, hepatitis C virus (HCV), hepatitis B virus (HBV) and cytomegalovirus (CMV)], the N-glycosylation profile of the antibodies is modified. Neu5Ac (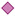), Gal (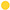), Man (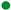), GlcNAc (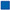), Fuc (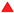). The arrows indicate an increase or decrease in the indicated glycan, and the triangles indicate an increase or decrease in the antibody function. (**C**) Changes in the N-glycosylation profile of antibodies in response to vaccination. Created in https://BioRender.com (accessed on 19 August 2025).

Changes in IgG Asn297-linked N-glycans have been reported during infection with *Mycobacterium tuberculosis* [69], and the glycosylation profiles of these antibodies can be used to distinguish active tuberculosis from latent tuberculosis. In a study conducted by Lu et al. [70] in two human cohorts from different geographic sites, it was found that IgGs from patients with active tuberculosis exhibit greater agalactosylation and greater amount of Fuc than individuals with latent tuberculosis, while individuals with latent tuberculosis exhibit more sialic acid compared to patients with active tuberculosis. In addition, IgGs from individuals with latent tuberculosis have increased ADCC and NK cell activation function; IgGs from these individuals may bind more efficiently to FcγRIIIa on NK cells, which correlates with improved ADCC. IgGs from individuals with active tuberculosis have lower ADCC function and a lower capacity to activate NK cells (Figure 5A) [70].

In another study, conducted by Grace et al. [71], the glycosylation profiles of IgGs from individuals with active tuberculosis, those with latent tuberculosis, and those who had completed treatment for active tuberculosis were compared. It was found that di-galactosylated IgGs (without sialic acid) are more frequent in individuals with latent tuberculosis and post-treatment and that mono-galactosylated, mono-sialylated and fucosylated IgGs are more frequent in patients with active tuberculosis than in individuals with latent tuberculosis and post-treatment. In addition, patients with active tuberculosis have higher titers of IgG4 that recognize the purified protein derivative (PPD) of *M. tuberculosis*, compared to individuals with latent tuberculosis and post-treatment. Furthermore, the antibodies of patients with active tuberculosis induce increased levels of antibody-dependent phagocytosis, compared to those of individuals with latent tuberculosis and those who have completed treatment [71].

When individuals infected with *M. tuberculosis* are co-infected with HIV, *M. tuberculosis*-specific antibody titers decrease, and the avidity of these antibodies against *M. tuberculosis* antigens (such as Ag85A) also decreases due to the reduction in T helper cells [5]. Macaques (*Macaca fascicularis*) respond similarly to humans to infection with *M. tuberculosis*, and when co-infected with *M. tuberculosis* and simian immunodeficiency virus (SIV), their *M. tuberculosis*-specific antibody titers decrease, and these antibodies have less interaction with FcγRIIa and FcγRIIIa, indicating a reduced ability to exert their effector functions. In addition, IgGs from co-infected macaques exhibit less fucosylation, more agalactosylation, and less di-galactosylation. This glycosylation profile correlates with bacterial load and pathological lesions, suggesting that it may serve as a biomarker of tuberculosis progression [69].

### 5.3. Modification of IgG N-Glycosylation in Viral Infections 

The N-glycosylation profile of IgGs is modified during infection with SARS-CoV-2. Fucosylation of anti-S IgG1 antibodies is lower in young patients compared to older patients. In addition, fucosylation undergoes more modifications during hospitalization in young patients compared to older patients. These modifications contribute to the change from a highly pro-inflammatory phenotype (afucosylated IgG) to a less pro-inflammatory phenotype (fucosylated IgG) in young people, which would allow the generation of an efficient response against the virus in the early phase of the disease in these people, compared to older people, where the response against the virus would be less balanced [72].

In another study of 159 patients infected with the SARS-CoV-2 virus, total IgG1 and anti-S-specific IgG1 from older individuals were found to have less galactosylation and sialylation than those of younger individuals. It was also observed that patients with severe COVID-19 have anti-S IgG1 with a higher presence of bisecting GlcNAc, lower galactosylation and lower sialylation compared to patients with mild or moderate COVID-19 [73].

Bournazos et al. demonstrated that, in the first days of SARS-CoV-2 infection, large amounts of anti-S IgG are produced, with low fucosylation and high galactosylation [43]. The low fucosylation of these antibodies promotes cellular activation through FcγRIII and favors the release of cytokines such as TNFα and IL-6 by macrophages, which correlates with a peak of inflammation at the time of seroconversion (Figure 5A). After days or weeks, the level of IgG fucosylation increases. These results suggest that the inflammatory function of afucosylated IgG could influence the pathology of COVID-19 [74]. 

Larsen et al. reported that, in general, IgG1s against membrane-embedded antigens (such as antigens from SARS-CoV-2 and other enveloped viruses) are afucosylated, while IgG1s against soluble antigens have high levels of fucosylation. Antibodies with low fucosylation activate FcγRIIIb-expressing NK cells, monocytes, macrophages, and granulocytes (Figure 5A), which is important for controlling infections and enhancing vaccine effectiveness but may also contribute to the pathology of some infections, as appears to be the case with SARS-CoV-2 [52]. Schwedler et al. corroborated these results, as they also observed that in the early stage of COVID-19, IgG antibodies have decreased fucosylation. In addition, they demonstrated that throughout the disease, there is a decrease in the galactosylation and sialylation of anti-S IgG1s [72].

In the first seven days of infection with the influenza virus, IgG has less fucosylation and bisecting GlcNAc and more sialylation. However, at 21 days, the glycosylation profile is different, since fucosylation increases and sialylation decreases, although the bisecting GlcNAc remains constant [75]. In contrast, in the case of HIV-1 infection, patients were found to have IgG antibodies with higher fucosylation and reduced levels of galactosylated and di-sialylated glycans; these antibodies are associated with lower ADCC (Figure 5B). The authors of this study corroborated that afucosylated mono- and di-sialylated antibodies are associated with increased ADCC [76]. When there is a co-infection of HIV with the hepatitis C virus, afucosylated IgG levels increase compared to the levels of these antibodies in separate infections with each virus [77].

During infection with dengue virus, non-neutralizing IgGs can aid in disease progression to hemorrhagic dengue by a process known as antibody-dependent enhancement (ADE) of infection, which involves opsonization of the virus by the non-neutralizing antibodies to increase virus internalization through FcγRs, inducing an increase in virions in infected cells [78]. It has been suggested that the production of IgGs that interact with greater affinity with activating FcγRs could predispose to ADE. Patients with hemorrhagic dengue have less fucosylation on antibodies specific to dengue virus antigens compared to patients who have febrile dengue, and this decrease in fucosylation induces an increase in the affinity of the antibodies for FcγRIIIa (as has been reported in other viral infections). Removing Gal from afucosylated antibodies decreases their ability to bind to FcγRIIa and FcγRIIIa [79]. These results suggest that the production of IgG with lower fucosylation and, therefore, a greater affinity for FcγRs, could favor ADE and the development of dengue hemorrhagic fever. Sastre et al. demonstrated that the ADE-inducing ability of afucosylated anti-dengue antibodies decreases when they are treated with an endo-beta-N-acetylglucosaminidase from *Corynebacterium diphtheriae*. This enzyme removes N-glycans from the Asn297 site of IgGs, making it a potential therapeutic option for the treatment of severe dengue [80]. Changes in the glycosylation profile of antibodies during some infections are summarized in Figure 5B.

## 6. Other Factors That Modify the N-Glycosylation of Antibodies

The N-glycosylation profile of antibodies is different in individuals from different geographic regions. For example, total IgGs from individuals in Africa show more agalactosylation, while those from individuals in the United States show more sialylation. These differences may be attributed to genetic variations, endemic infections, environmental factors, and dietary differences [81]. The N-glycosylation profile of antibodies also changes with age. The first IgGs produced by children are agalactosylated, and by age 25, galactosylation begins to increase until the level of mono-galactosylated N-glycans remains stable [82]. From the age of 40, the level of galactosylation of IgGs decreases [83]. The presence of IgG with fucosylated and digalactosylated biantennary glycans has been associated with aging in older people (mean age, 58–64 years) [84]. On the other hand, the antibodies produced by pregnant women against influenza virus type 1 hemagglutinin have a greater capacity to bind to FcγRI and higher avidity, suggesting that their N-glycosylation profile is different [85]. Finally, IgA with aberrant N-glycosylation has been found in the plasma of patients with ovarian cancer, breast cancer, colorectal cancer and liver cancer related to hepatitis B virus [86,87,88]. 

The glycosylation profile of antibodies is also modified by some activators of the immune system. Oligodeoxynucleotide CpG (a ligand of TLR9) and IL-21 (a cytokine produced by T cells) increase galactosylation and reduce bisecting GlcNAc levels in IgG1 Fc, while vitamin A significantly reduces galactosylation and sialylation levels [89]. When B cells are stimulated with IFNγ, agalactosylation levels of IgGs decrease, and the frequency of mono-galactosylation and di-galactosylation increases; these effects are due to the upregulation of β-1,4-galactosyltransferase [89,90]. After stimulation with IL-21 and IL-17A, sialylation increases because of the upregulation of β-galactosidase α-2,6-sialyltransferase 1 [90]. However, stimulation with IL-4, IL-6, TNFα, TGFβ, and lymphotoxin-α had no effect on IgG N-glycosylation [89].

## 7. Vaccination-Induced Antibody N-Glycosylation Profiles

The development of a protective vaccine against any infection requires the induction of highly neutralizing antibodies [91]. A long-lasting B-cell memory and plasma-cell response should also be induced. IgGs are important mediators of vaccine-induced immunity by performing effector functions, such as complement activation and cellular activation by FcRs [92]. In humans, vaccination induces changes in the N-glycosylation profile of IgGs, which may activate different cells or confer different functions. These changes in the N-glycosylation profile are dependent on the antigen, the vaccine platform and adjuvant used, as well as the individuals’ age. Mahan et al. demonstrated that vaccine-induced changes in the N-glycosylation profile are restricted to the vaccine-induced population of specific antibodies and do not affect the overall humoral immune response. In addition, the changes in the N-glycosylation profile of IgGs induced by vaccines are similar, even if the vaccine is applied in different geographical regions [81].

Tetanus and influenza (H1N1 and seasonal) vaccines induce a transient increase in galactosylation and sialylation, a decrease in the frequency of bisecting GlcNAc, and an increase in the amount of sialic acid per Gal following vaccination (Figure 5C) [93]. Wang et al. observed similar effects after vaccination against H1N1 influenza: sialylated and fucosylated glycoforms were elevated, peaking at seven days post-vaccination, but these levels decreased at 30 days [94]. In this study, they demonstrated that the increase in sialylated and fucosylated glycoforms is due to the increased expression of ST6GAL1 and FUT8 in plasmablasts and B cells. In addition, it was observed that these two enzymes are more expressed in plasmablasts than in memory B cells, suggesting that the production of sialylated and fucosylated N-glycans is carried out by plasmablasts, while the production of less sialylated and less fucosylated N-glycans is carried out by memory B cells [94]. After influenza vaccination, anti-hemagglutinin IgGs with higher sialylation have a higher affinity for their antigen. If immune complexes formed by sialylated antibodies are used to immunize mice, it is observed that the anti-hemagglutinin IgGs that are induced by these immune complexes have greater affinity for hemagglutinin, compared to IgGs induced by immune complexes formed by asialylated antibodies. Additionally, these immune complexes induce the upregulation of FcγRIIb on B cells [94].

Adenovirus and protein-based HIV vaccines induce antibodies with an inflammatory N-glycosylation profile, which is characterized by low galactosylation and sialylation, increased fucosylation, and increased presence of bisecting GlcNAc; increased bisecting GlcNAc is related to increased ADCC. In addition, the adenovirus-based vaccine induces greater sialylation and a higher presence of bisecting GlcNAc compared to the protein vaccine [81]. IgG1s specific against enveloped antigens, capable of neutralizing HIV, are more sialylated than non-neutralizing IgG1s (Figure 5C). If mice are immunized with immune complexes, it is observed that sialylated immune complexes distribute more rapidly to follicular dendritic cells in the germinal centers, so these immune complexes favor the induction of B cells with high-affinity receptors [91]. 

Regarding SARS-CoV-2 vaccines, several studies have been conducted with different vaccine platforms. Pfizer-BioNTech BNT162b2 and Moderna mRNA-1273 mRNA-based vaccines, and AstraZeneca ChAdOx1 nCoV-19 and Janssen Ad26.CoV2.S adenovirus-based vaccines, induce the production of anti-S IgG and IgA antibodies with neutralizing capacity, and protect against severe forms of COVID-19, especially if people have a history of SARS-CoV-2 infection [95,96]. All these vaccines induce IgG antibodies with a transient decrease in their fucosylation, which increases about two weeks after vaccination in most vaccines, except in the case of the Janssen vaccine, in which a sustained trend of afucosylation was observed after 50 days (Figure 5C) [95,96,97]. Afucosylated IgG1s specific for SARS-CoV-2 have a high affinity for FcγRIII, and the presence of afucosylated IgGs correlates with increased pro-inflammatory cytokines in COVID-19 patients but not in vaccinated individuals [96]. The Pfizer and Janssen vaccines induce a significant increase in galactosylation and sialylation of anti-S IgG1 and a greater presence of bisecting GlcNAc in these antibodies [96]. These differences in the N-glycosylation profile may be due to the adjuvant effect of the different vaccine platforms.

Bartsch et al. evaluated the effect of different adjuvants on the N-glycosylation profile of antibodies induced by ovalbumin vaccination. They tested Freund’s complete adjuvant (containing 5 mg/mL of *M. tuberculosis* H37Ra), Montanide adjuvant, aluminum hydroxide, Adju-Phos adjuvant (aluminum phosphate), AddaVax adjuvant (squalene-based oil-in-water nano-emulsion), and TLRs agonists lipopolysaccharide, mono-phosphoryl lipid A, R848, and poly (I:C). They found that these adjuvants induce IgG with high levels of galactosylation and sialylation at 7 days post-vaccination. However, at day 14 post-vaccination, lower sialylation of IgG was observed when Freund’s complete adjuvant was used, and this adjuvant downregulates the expression of ST6GAL1 in germinal center B cells, through a mechanism that depends on IL-6 produced by TFH cells [98].

In mice, vaccination against SIV is more efficient if an aluminum adjuvant is used, compared to MF59 (oil-in-water emulsion) adjuvant, and the IgGs that are induced with the aluminum adjuvant are less sialylated and galactosylated. The potential of these vaccines to induce a germinal center antibody and B-cell response is dependent on IFNγ- and IL-17-producing TFH cells, and the presence of these cells correlates with lower levels of IgG sialylation and galactosylation [99].

## 8. Importance of N-Glycosylation in Monoclonal Antibodies for Therapeutic Use

In 1986, the FDA approved muromonab-CD3, the first therapeutic monoclonal antibody for use in humans [100]. Since then, monoclonal antibodies have become the most versatile group of drugs, with more than 200 monoclonal antibodies approved internationally to date, 61 of them since 2016 [101,102]. The monoclonal antibody market reached USD $252.6 billion in 2024 and, with a compound annual growth rate of 14.5%, is expected to reach a value of USD $497.5 billion by 2029 [103].

The success of monoclonal antibodies is mainly due to their exquisite specificity for their therapeutic target, which improves their safety profile compared to drugs obtained by chemical synthesis and makes them the closest prototype to Paul Ehrlich’s concept of a magic bullet. Since the commercialization of muromonab-CD3, a murine antibody used to prevent renal allograft rejection [104], the biopharmaceutical industry has improved the design of monoclonal antibodies to obtain fully human sequences in different formats in addition to IgG, such as bispecific antibodies, antibody-drug conjugates and antibody fragments. Currently, 70% of commercial monoclonal antibodies are IgG1 and are obtained from Chinese hamster ovary (CHO) cell cultures, as these cells offer several advantages: expression yields of up to 10 g/L, robust cell growth, effective post-translational modifications, and well-established standards to meet good manufacturing practices [105,106,107]. 

Like any pharmaceutical product, monoclonal antibodies must demonstrate their efficacy, safety and quality in order to obtain a sanitary registration for use in humans. In this sense, N-glycosylation is the critical quality attribute that most influences the efficacy and safety of monoclonal antibodies, specifically in the intensity of their effector functions, their persistence in the bloodstream and their immunogenicity [108]. Monoclonal antibodies are artificial molecules whose heterogeneity comes mainly from the galactosylation, fucosylation and sialylation of biantennary complex oligosaccharides [106]. The most abundant glycoforms of monoclonal antibodies are fucosylated structures with none, one, or two Gal [109]. Most monoclonal antibodies contain the characteristic N-glycosylation site at Asn297 in the CH2 region, but some humanized or chimeric antibodies, such as cetuximab, may have an additional glycosylation site at Asn88 in the VH region [110,111]. Compared to monoclonal antibodies, natural human IgGs have higher levels of biantennary glycans and sialic acid, almost no high-Man oligosaccharides or aglycosylated variants (these represent less than 0.1% and 0.2%, respectively), and no N-glycolylneuraminic acid (Neu5Gc, one of the two types of sialic acid) or α-1,3 Gal [109]. As mentioned above, the α-2,6-terminal sialic acid of the Fc fragment of natural human IgGs has an anti-inflammatory function [54]. This anti-inflammatory function is associated with the presence of sialic acid in 2,6 linkage, but not in 2,3 linkage, to the penultimate Gal of the oligosaccharide [112]. The differential function could be explained by the fact that changing the glycosidic bond from the 2,6 to the 2,3 position alters the flexibility and the shape of oligosaccharide [113]. Some of the non-human cell lines in which monoclonal antibodies are produced lack ST6GAL1 and generate monoclonal antibodies with only α-2,3-sialylation. Genetic modification of these cell lines can lead to the production of monoclonal antibodies with α-2,6-sialylation, which more closely resembles the glycosylation pattern of natural human IgGs [114,115].

### 8.1. Effect of N-Glycosylation on the Pharmacokinetic Profile of Monoclonal Antibodies

One of the most important studies for the development of monoclonal antibodies is the determination of their pharmacokinetic profile, which describes the changes in plasma concentrations of the monoclonal antibody as a function of time and the influence of these changes on its therapeutic effect [116]. Monoclonal antibodies exhibit similar pharmacokinetic behaviors compared to each other and to endogenous antibodies, and this profile is influenced by the biodistribution of their therapeutic target and by FcRs, including the neonatal Fc receptor (FcRn), which is highly expressed in placenta, liver, intestine, and capillary endothelial cells vessels [110,117,118,119]. The pharmacokinetic parameters of an IgG1 monoclonal antibody are as follows: clearance from the central compartment (CL) = 0.15 mL/h/kg; volume of the central compartment (V1) = 46.31 mL/kg; inter-compartment distribution clearance (Q) = 0.27 mL/h/kg and volume of the peripheral compartment (V2) = 31.47 mL/kg [119]. Other typical pharmacokinetic parameters are as follows: maximum plasma concentration time (Tmax) after subcutaneous or intramuscular administration = 1–8 days; bioavailability after subcutaneous or intramuscular administration = 50–100%, and elimination half-life = 21 days [110,119]. However, these pharmacokinetic parameters can be altered due to the glycosylation profile and, consequently, the processes of antibody absorption, distribution, metabolism and excretion can also be modified. 

Absorption refers to the passage of a drug from the site of administration into the bloodstream. In the case of monoclonal antibodies, most are administered intravenously and, to a lesser extent, subcutaneously or intramuscularly. In the intravenous route, the total dose administered reaches the bloodstream directly, while in the subcutaneous and intramuscular routes, the monoclonal antibodies pass into the bloodstream through the lymphatic system, which allows the transport of molecules of more than 20 kDa through the convective flow of interstitial fluids [110]. The absorption of a drug depends on its net charge, and some carbohydrates, such as the negatively charged sialic acid, can modify the net charge of an antibody [120].

Distribution refers to the dispersion of a drug from the bloodstream to the rest of the tissues of a living organism. In the case of monoclonal antibodies, a crucial mechanism of distribution is recycling through FcRn; however, this binding is independent of Fc N-glycosylation [121]. On the other hand, monoclonal antibodies are distributed through vascular endothelial cells by convection, except in the brain, which is protected by the blood–brain barrier [110]. Additionally, monoclonal antibodies are also distributed and accumulated depending on the location of their antigen and of FcRs, so N-glycosylation profiles that affect the binding of monoclonal antibodies to FcRs (such as those described in Section 8.2) could also affect the distribution of the monoclonal antibody. 

The metabolism of monoclonal antibodies includes deglycosylation by glycosidases that cleave the glycosidic bonds of the antibody glycans [121], and hydrolysis of the light and heavy chains to peptides and subsequently to amino acids in all tissues and plasma. These amino acids are reused to form new proteins. The skin, muscle, liver, and gut are estimated to contribute 33%, 24%, 16%, and 12% of the percentage of monoclonal antibody metabolism, respectively [122]. Monoclonal antibodies are very large structures that cannot be filtered in the renal glomerulus; therefore, their elimination from the circulation is mediated by receptors [121]. Monoclonal antibodies with high Man content are removed from the circulation by the Man receptor, which recognizes oligosaccharides with Man or with GlcNAc and is highly expressed on cells of the immune system [109,110,121]. The other receptor by which monoclonal are removed is the asialoglycoprotein receptor, which is found in the liver and recognizes terminal galactosylation [121,123]. In both cases, monoclonal antibodies that have high Man, GlcNAc or Gal will have a higher elimination rate and, consequently, a shorter half-life in circulation. In the case of aglycosylated monoclonal antibodies, it has been observed that they can have conformational changes, lower thermal stability and a greater tendency to aggregation. However, in humans, aglycosylated monoclonal antibodies have a half-life equal to that of glycosylated monoclonal antibodies [109,110]. 

The absorption, distribution and elimination of a drug depend on its net charge and, as mentioned above, some carbohydrates can modify the net charge of an antibody [120]. Antibodies with a net negative charge have limited extravasation rates because of charge repulsion with cell membranes, but higher rates of distribution and clearance are expected in antibodies with a net positive charge [124]. 

### 8.2. Effect of N-Glycosylation on the Pharmacodynamic Profile of Monoclonal Antibodies

The main carbohydrates that affect the pharmacodynamic profile of therapeutic monoclonal antibodies are terminal Gal, Fuc, Man and sialic acid. Galactosylation does not affect the charge or hydrophobicity of a monoclonal antibody, but it may increase its complement-dependent cytotoxicity by increasing its affinity for C1q. In contrast, galactosylation has little or no effect on ADCC [106,109,121]. Fuc does not affect the charge or hydrophobicity of a monoclonal antibody, but its absence increases ADCC because it increases the affinity of the monoclonal antibody for FcγRIIIa [109]. IgG monoclonal antibodies with a high Man content have an increase in their binding to FcγRs and thus increase their ADCC. In contrast, these oligosaccharides reduce C1q-mediated activity [109]. The presence of sialic acid may negatively affect ADCC and complement-dependent cytotoxicity, and it can also decrease the monoclonal antibody binding to FcγRIIIa on NK cells in vitro [109,121]. 

### 8.3. Effect of N-Glycosylation on the Safety Profile of Monoclonal Antibodies

Immunogenicity is the main safety concern for any biotherapeutic, including monoclonal antibodies. It can be defined as the induction of a cellular and humoral immune response, typically characterized by the production of anti-drug antibodies by the patient’s immune system [125]. Once induced, anti-drug antibodies interact with the monoclonal antibody and decrease its efficacy. The anti-drug antibodies/monoclonal antibody complexes can induce a transient and subclinical effect, or cause symptoms that range from mild or moderate (skin rash, fever, urticaria, hypotension, serum sickness) to severe anaphylaxis [125,126]. The N-glycosylation profile can affect the immunogenicity of a monoclonal antibody. Since monoclonal antibodies are produced in non-human cell lines, they are expected to have non-human N-glycans. Neu5Gc and α-1,3 Gal are not found in natural human IgGs, but they are found in monoclonal antibodies produced in murine cell lines, especially in the Fab, and they have been linked to immunogenicity events [109]. In addition, N-glycan heterogeneity and complexity are associated with higher risk of immunogenicity. Some commercial monoclonal antibodies have up to 10% of high Man content, and this promotes their endocytosis by macrophages and dendritic cells through the Man receptor, leading to more efficient antigen processing and presentation, but also to higher immunogenicity concerns [127]. 

The studies summarized in this section indicate that the glycosylation profile is a critical parameter of therapeutic monoclonal antibodies. Currently, there is a wide field of research regarding the control and optimization of the glycosylation profile of a monoclonal antibody, in order to modify its pharmacokinetic, pharmacodynamic and safety profiles [108]. 

## 9. Concluding Remarks

Currently, there is a growing body of evidence about the biological effects of antibody N-glycosylation, mainly on IgG antibodies, but also on the other antibody classes. N-glycosylation modifies the binding of antibodies to their FcRs, and also their ability to activate complement. In addition, the N-glycans of IgA can inhibit bacterial adhesion and neutralize some viruses independently of Fab recognition. During infections, N-glycosylation can modulate the immune response towards an anti-inflammatory or pro-inflammatory profile. However, these effects have only been studied in some diseases that are considered major health problems, such as COVID-19, tuberculosis and HIV infection, but remain unknown in other infections. N-glycosylation profiles are modified during infections, after infection treatment and after vaccination. The underlying mechanisms that lead to these modifications have not been elucidated, but they could include signaling pathways and extracellular signals that change the expression levels and/or the activity of the many glycosyltransferases and glycosidases that remodel the carbohydrate–protein complexes. Further studies about antibody N-glycosylation could lead to new therapeutic antibodies with specific N-glycosylation profiles, tailored to the infecting microorganism and to the infection stage, in order to ensure a more effective infection control. In addition, a better understanding of the biological effects of antibody N-glycosylation would be useful to increase the effectiveness and safety of therapeutic monoclonal antibodies in general.

## Data Availability

No new data were created or analyzed in this study. Data sharing is not applicable to this article.

## References

[1] Damelang T., Brinkhaus M., van Osch T.L.J., Schuurman J., Labrijn A.F., Rispens T., Vidarsson G. (2023). Impact of Structural Modifications of IgG Antibodies on Effector Functions. Front. Immunol..

[2] Pan S., Manabe N., Yamaguchi Y. (2021). 3D Structures of Iga, Igm, and Components. Int. J. Mol. Sci..

[3] Kuijpers T. (2014). Fc-Dependent Mechanisms of Action: Roles of FcγR and FcRn. Clin. Exp. Immunol..

[4] Bruhns P., Iannascoli B., England P., Mancardi D.A., Fernandez N., Jorieux S., Daë Ron M. (2009). Specificity and Affinity of Human Fc Receptors and Their Polymorphic Variants for Human IgG Subclasses. Blood.

[5] McLean M.R., Lu L.L., Kent S.J., Chung A.W. (2019). An Inflammatory Story: Antibodies in Tuberculosis Comorbidities. Front. Immunol..

[6] Napodano C., Marino M.P., Stefanile A., Pocino K., Scatena R., Gulli F., Rapaccini G.L., Delli Noci S., Capozio G., Rigante D. (2021). Immunological Role of IgG Subclasses. Immunol. Investig..

[7] Rispens T., Huijbers M.G. (2023). The Unique Properties of IgG4 and Its Roles in Health and Disease. Nat. Rev. Immunol..

[8] Ding L., Chen X., Cheng H., Zhang T., Li Z. (2022). Advances in IgA Glycosylation and Its Correlation with Diseases. Front. Chem..

[9] Jones K., Savulescu A.F., Brombacher F., Hadebe S. (2020). Immunoglobulin M in Health and Diseases: How Far Have We Come and What Next?. Front. Immunol..

[10] Haslund-Gourley B., Woloszcuk K., Hou J., Connors J., Cusimano G., Bell M., Taramangalam B., Fourati S., Mege N., Bernui M. (2023). IgM N-Glycosylation Correlates with COVID-19 Severity and Rate of Complement Deposition. Nat. Commun..

[11] Arnold J.N., Radcliffe C.M., Wormald M.R., Royle L., Harvey D.J., Crispin M., Dwek R.A., Sim R.B., Rudd P.M. (2004). The Glycosylation of Human Serum IgD and IgE and the Accessibility of Identified Oligomannose Structures for Interaction with Mannan-Binding Lectin1. J. Immunol..

[12] Itoh N., Ohshima Y. (2023). The Dual Aspects of IgD in the Development of Tolerance and the Pathogenesis of Allergic Diseases. Allergol. Int..

[13] Shade K.T.C., Conroy M.E., Washburn N., Kitaoka M., Huynh D.J., Laprise E., Patil S.U., Shreffler W.G., Anthony R.M. (2020). Sialylation of Immunoglobulin E Is a Determinant of Allergic Pathogenicity. Nature.

[14] Plattner K., Bachmann M.F., Vogel M. (2023). On the Complexity of IgE: The Role of Structural Flexibility and Glycosylation for Binding Its Receptors. Front. Allergy.

[15] Nimmerjahn F., Vidarsson G., Cragg M.S. (2023). Effect of Posttranslational Modifications and Subclass on IgG Activity: From Immunity to Immunotherapy. Nat. Immunol..

[16] Ramakrishnan P. (2024). O-GlcNAcylation and Immune Cell Signaling: A Review of Known and a Preview of Unknown. J. Biol. Chem..

[17] Schjoldager K.T., Narimatsu Y., Joshi H.J., Clausen H. (2020). Global View of Human Protein Glycosylation Pathways and Functions. Nat. Rev. Mol. Cell Biol..

[18] Freeze H.H., Boyce M., Zachara N.E., Hart G.W., Schnaar R.L. (2022). Glycosylation Precursors. Essentials of Glycobiology.

[19] Jiménez del Val I., Constantinou A., Dell A., Haslam S., Polizzi K.M., Kontoravdi C. (2013). A Quantitative and Mechanistic Model for Monoclonal Antibody Glycosylation as a Function of Nutrient Availability during Cell Culture. BMC Proc..

[20] Wang T.T. (2019). IgG Fc Glycosylation in Human Immunity. Current Topics in Microbiology and Immunology.

[21] Hao C., Zou Q., Bai X., Shi W. (2025). Effect of Glycosylation on Protein Folding: From Biological Roles to Chemical Protein Synthesis. iScience.

[22] Trzos S., Link-Lenczowski P., Pocheć E. (2023). The Role of N-Glycosylation in B-Cell Biology and IgG Activity. The Aspects of Autoimmunity and Anti-Inflammatory Therapy. Front. Immunol..

[23] Reily C., Stewart T.J., Renfrow M.B., Novak J. (2019). Glycosylation in Health and Disease. Nat. Rev. Nephrol..

[24] Stanley P., Moremen K.W., Lewis N.E., Taniguchi N., Aebi M. (2022). N-Glycans. Encyclopedia of Cell Biology: Volume 1–6.

[25] Hayes J.M., Cosgrave E.F.J., Struwe W.B., Wormald M., Davey G.P., Jefferis R., Rudd P.M. (2014). Glycosylation and Fc Receptors. Curr. Top. Microbiol. Immunol..

[26] Esmail S., Manolson M.F. (2021). Advances in Understanding N-Glycosylation Structure, Function, and Regulation in Health and Disease. Eur. J. Cell Biol..

[27] Irvine E.B., Alter G. (2021). Understanding the Role of Antibody Glycosylation through the Lens of Severe Viral and Bacterial Diseases. Glycobiology.

[28] Vidarsson G., Dekkers G., Rispens T. (2014). IgG Subclasses and Allotypes: From Structure to Effector Functions. Front. Immunol..

[29] Krištić J., Lauc G. (2024). The Importance of IgG Glycosylation—What Did We Learn after Analyzing over 100,000 Individuals. Immunol. Rev..

[30] Hui G.K., Wright D.W., Vennard O.L., Rayner L.E., Pang M., Yeo S.C., Gor J., Molyneux K., Barratt J., Perkins S.J. (2015). The Solution Structures of Native and Patient Monomeric Human IgA1 Reveal Asymmetric Extended Structures: Implications for Function and IgAN Disease. Biochem. J..

[31] Lombana T.N., Rajan S., Zorn J.A., Mandikian D., Chen E.C., Estevez A., Yip V., Bravo D.D., Phung W., Farahi F. (2019). Production, Characterization, and In Vivo Half-Life Extension of Polymeric IgA Molecules in Mice. mAbs.

[32] Steffen U., Koeleman C.A., Sokolova M.V., Bang H., Kleyer A., Rech J., Unterweger H., Schicht M., Garreis F., Hahn J. (2020). IgA Subclasses Have Different Effector Functions Associated with Distinct Glycosylation Profiles. Nat. Commun..

[33] Shade K.T.C., Platzer B., Washburn N., Mani V., Bartsch Y.C., Conroy M., Pagan J.D., Bosques C., Mempel T.R., Fiebiger E. (2015). A Single Glycan on IgE Is Indispensable for Initiation of Anaphylaxis. J. Exp. Med..

[34] Mellis S.J., Baenziger J.U. (1983). Structures of the Oligosaccharides Present at the Three Asparagine-Linked Glycosylation Sites of Human IgD. J. Biol. Chem..

[35] Chen J., Fang M., Chen X., Yi C., Ji J., Cheng C., Wang M., Gu X., Sun Q., Gao C. (2017). N-Glycosylation of Serum Proteins for the Assessment of Patients with IgD Multiple Myeloma. BMC Cancer.

[36] Van De Bovenkamp F.S., Derksen N.I.L., Ooijevaar-de Heer P., Van Schie K.A., Kruithof S., Berkowska M.A., Ellen van der Schoot C., IJspeert H., Van Der Burg M., Gils A. (2018). Adaptive Antibody Diversification through N-Linked Glycosylation of the Immunoglobulin Variable Region. Proc. Natl. Acad. Sci. USA.

[37] Van de Bovenkamp F.S., Derksen N.I.L., van Breemen M.J., de Taeye S.W., Ooijevaar-de Heer P., Sanders R.W., Rispens T. (2018). Variable Domain N-Linked Glycans Acquired during Antigen-Specific Immune Responses Can Contribute to Immunoglobulin G Antibody Stability. Front. Immunol..

[38] Huang T., Chen X., Zhao C., Liu X., Zhang Z., Li T., Sun R., Gu H., Gu J. (2016). Sialylated Immunoglobulin G Can Neutralize Influenza Virus Infection through Receptor Mimicry. Oncotarget.

[39] Thaysen-Andersen M., Packer N.H. (2012). Site-Specific Glycoproteomics Confirms That Protein Structure Dictates Formation of N-Glycan Type, Core Fucosylation and Branching. Glycobiology.

[40] Ahmed A.A., Giddens J., Pincetic A., Lomino J.V., Ravetch J.V., Wang L.X., Bjorkman P.J. (2014). Structural Characterization of Anti-Inflammatory Immunoglobulin G Fc Proteins. J. Mol. Biol..

[41] Shade K.T., Conroy M.E., Anthony R.M. (2019). IgE Glycosylation in Health and Disease. Current Topics in Microbiology and Immunology.

[42] Zlatina K., Galuska S.P. (2021). Immunoglobulin Glycosylation—An Unexploited Potential for Immunomodulatory Strategies in Farm Animals. Front. Immunol..

[43] Bournazos S., Gupta A., Ravetch J.V. (2020). The Role of IgG Fc Receptors in Antibody-Dependent Enhancement. Nat. Rev. Immunol..

[44] Kao D., Danzer H., Collin M., Groß A., Eichler J., Stambuk J., Lauc G., Lux A., Nimmerjahn F. (2015). A Monosaccharide Residue Is Sufficient to Maintain Mouse and Human IgG Subclass Activity and Directs IgG Effector Functions to Cellular Fc Receptors. Cell Rep..

[45] Van Coillie J., Schulz M.A., Bentlage A.E.H., de Haan N., Ye Z., Geerdes D.M., van Esch W.J.E., Hafkenscheid L., Miller R.L., Narimatsu Y. (2022). Role of N-Glycosylation in FcγRIIIa Interaction with IgG. Front. Immunol..

[46] Falconer D.J., Subedi G.P., Marcella A.M., Barb A.W. (2018). Antibody Fucosylation Lowers the FcγRIIIa/CD16a Affinity by Limiting the Conformations Sampled by the N162-Glycan. ACS Chem. Biol..

[47] Shinkawa T., Nakamura K., Yamane N., Shoji-Hosaka E., Kanda Y., Sakurada M., Uchida K., Anazawa H., Satoh M., Yamasaki M. (2003). The Absence of Fucose but Not the Presence of Galactose or Bisecting N-Acetylglucosamine of Human IgG1 Complex-Type Oligosaccharides Shows the Critical Role of Enhancing Antibody-Dependent Cellular Cytotoxicity. J. Biol. Chem..

[48] Dekkers G., Treffers L., Plomp R., Bentlage A.E.H., de Boer M., Koeleman C.A.M., Lissenberg-Thunnissen S.N., Visser R., Brouwer M., Mok J.Y. (2017). Decoding the Human Immunoglobulin G-Glycan Repertoire Reveals a Spectrum of Fc-Receptor- and Complement-Mediated-Effector Activities. Front. Immunol..

[49] Larsen M.D., Lopez-Perez M., Dickson E.K., Ampomah P., Tuikue Ndam N., Nouta J., Koeleman C.A.M., Ederveen A.L.H., Mordmüller B., Salanti A. (2021). Afucosylated Plasmodium Falciparum-Specific IgG Is Induced by Infection but Not by Subunit Vaccination. Nat. Commun..

[50] Ackerman M.E., Crispin M., Yu X., Baruah K., Boesch A.W., Harvey D.J., Dugast A.S., Heizen E.L., Ercan A., Choi I. (2013). Natural Variation in Fc Glycosylation of HIV-Specific Antibodies Impacts Antiviral Activity. J. Clin. Investig..

[51] Bournazos S., Vo H.T.M., Duong V., Auerswald H., Ly S., Sakuntabhai A., Dussart P., Cantaert T., Ravetch J.V. (2021). Antibody Fucosylation Predicts Disease Severity in Secondary Dengue Infection. Science.

[52] Larsen M.D., de Graaf E.L., Sonneveld M.E., Plomp H.R., Nouta J., Hoepel W., Chen H.J., Linty F., Visser R., Brinkhaus M. (2021). Afucosylated IgG Characterizes Enveloped Viral Responses and Correlates with COVID-19 Severity. Science.

[53] Shields R.L., Lai J., Keck R., O’Connell L.Y., Hong K., Gloria Meng Y., Weikert S.H.A., Presta L.G. (2002). Lack of Fucose on Human IgG1 N-Linked Oligosaccharide Improves Binding to Human FcγRIII and Antibody-Dependent Cellular Toxicity. J. Biol. Chem..

[54] Kaneko Y., Nimmerjahn F., Ravetch J. (2006). V Anti-Inflammatory Activity of Immunoglobulin G Resulting from Fc Sialylation. Science.

[55] Anthony R.M., Kobayashi T., Wermeling F., Ravetch J.V. (2011). Intravenous Gammaglobulin Suppresses Inflammation through a Novel T H 2 Pathway. Nature.

[56] Vattepu R., Sneed S.L., Anthony R.M. (2022). Sialylation as an Important Regulator of Antibody Function. Front. Immunol..

[57] Gao C., Chen Q., Hao X., Wang Q. (2023). Immunomodulation of Antibody Glycosylation through the Placental Transfer. Int. J. Mol. Sci..

[58] Van Gool M.M.J., van Egmond M. (2020). IgA and FcαRI: Versatile Players in Homeostasis, Infection, and Autoimmunity. Immunotargets Ther..

[59] Gomes M.M., Wall S.B., Takahashi K., Novak J., Renfrow M.B., Herr A.B. (2008). Analysis of IgA1 N-Glycosylation and Its Contribution to FcαRI Binding. Biochemistry.

[60] Göritzer K., Turupcu A., Maresch D., Novak J., Altmann F., Oostenbrink C., Obinger C., Strasser R. (2019). Distinct Fcα Receptor N-Glycans Modulate the Binding Affinity to Immunoglobulin A (IgA) Antibodies. J. Biol. Chem..

[61] Colucci M., Stöckmann H., Butera A., Masotti A., Baldassarre A., Giorda E., Petrini S., Rudd P.M., Sitia R., Emma F. (2015). Sialylation of N-Linked Glycans Influences the Immunomodulatory Effects of IgM on T Cells. J. Immunol..

[62] Wei B., Gao X., Cadang L., Izadi S., Liu P., Zhang H.M., Hecht E., Shim J., Magill G., Pabon J.R. (2021). Fc Galactosylation Follows Consecutive Reaction Kinetics and Enhances Immunoglobulin G Hexamerization for Complement Activation. mAbs.

[63] Malhotra R., Wormald M., Rudd P., Fischer P., Dwek R., Sim R. (1995). Glycosylation Changes of IgG Associated with Rheumatooid Arthritis Can Activate Complement via the Mannose-Binding Protein. Nat. Med..

[64] Royle L., Roos A., Harvey D.J., Wormald M.R., Van Gijlswijk-Janssen D., Redwan E.R.M., Wilson I.A., Daha M.R., Dwek R.A., Rudd P.M. (2003). Secretory IgA N- and O-Glycans Provide a Link between the Innate and Adaptive Immune Systems. J. Biol. Chem..

[65] Goonatilleke E., Smilowitz J.T., Mariño K.V., German B.J., Lebrilla C.B., Barboza M. (2019). Immunoglobulin A N-Glycosylation Presents Important Body Fluid-Specific Variations in Lactating Mothers. Mol. Cell. Proteom..

[66] Schroten H., Stapper C., Plogmann R., Köhler H., Köhler K., Jö J., Hacker J., Hanisch F.-G. (1998). Fab-Independent Antiadhesion Effects of Secretory Immunoglobulin A on S-Fimbriated Escherichia Coli Are Mediated by Sialyloligosaccharides. Infect. Immun..

[67] Mathias A., Corthésy B. (2011). N-Glycans on Secretory Component: Mediators of the Interaction between Secretory IgA and Gram-Positive Commensals Sustaining Intestinal Homeostasis. Gut Microbes.

[68] Maurer M.A., Meyer L., Bianchi M., Turner H.L., Le N.P.L., Steck M., Wyrzucki A., Orlowski V., Ward A.B., Crispin M. (2018). Glycosylation of Human IgA Directly Inhibits Influenza A and Other Sialic-Acid-Binding Viruses. Cell Rep..

[69] Haycroft E.R., Damelang T., Lopez E., Rodgers M.A., Wines B.D., Hogarth M., Ameel C.L., Kent S.J., Scanga C.A., O’Connor S.L. (2023). Antibody Glycosylation Correlates with Disease Progression in SIV-Mycobacterium Tuberculosis Coinfected Cynomolgus Macaques. Clin. Transl. Immunol..

[70] Lu L.L., Chung A.W., Rosebrock T.R., Ghebremichael M., Yu W.H., Grace P.S., Schoen M.K., Tafesse F., Martin C., Leung V. (2016). A Functional Role for Antibodies in Tuberculosis. Cell.

[71] Grace P.S., Dolatshahi S., Lu L.L., Cain A., Palmieri F., Petrone L., Fortune S.M., Ottenhoff T.H.M., Lauffenburger D.A., Goletti D. (2021). Antibody Subclass and Glycosylation Shift Following Effective TB Treatment. Front. Immunol..

[72] Schwedler C., Grzeski M., Kappert K., Rust J., Heymann G., Hoppe B., Blanchard V. (2022). Coronavirus Disease 2019-Related Alterations of Total and Anti-Spike IgG Glycosylation in Relation to Age and Anti-Spike IgG Titer. Front. Microbiol..

[73] Pongracz T., Nouta J., Wang W., Van Meijgaarden K.E., Linty F., Vidarsson G., Joosten S.A., Ottenhoff T.H.M., Hokke C.H., De Vries J.J.C. (2022). Immunoglobulin G1 Fc Glycosylation as an Early Hallmark of Severe COVID-19. eBioMedicine.

[74] Hoepel W., Chen H.-J., Geyer C.E., Allahverdiyeva S., Manz X.D., de Taeye S.W., Aman J., Mes L., Steenhuis M., Griffith G.R. (2021). High Titers and Low Fucosylation of Early Human Anti-SARS-CoV-2 IgG Promote Inflammation by Alveolar Macrophages. Sci. Transl. Med..

[75] Kljaković-Gašpić Batinjan M., Petrović T., Vučković F., Hadžibegović I., Radovani B., Jurin I., Đerek L., Huljev E., Markotić A., Lukšić I. (2023). Differences in Immunoglobulin G Glycosylation Between Influenza and COVID-19 Patients. Engineering.

[76] Vadrevu S.K., Trbojevic-Akmacic I., Kossenkov A.V., Colomb F., Giron L.B., Anzurez A., Lynn K., Mounzer K., Landay A.L., Kaplan R.C. (2018). Frontline Science: Plasma and Immunoglobulin G Galactosylation Associate with HIV Persistence during Antiretroviral Therapy. J. Leukoc. Biol..

[77] Giron L.B., Azzoni L., Yin X., Lynn K.M., Ross B.N., Fair M., Damra M., Sciorillo A.C., Liu Q., Jacobson J.M. (2020). Hepatitis C Virus Modulates IgG Glycosylation in HIV Co-Infected Antiretroviral Therapy Suppressed Individuals. AIDS.

[78] Endy T.P., Nisalak A., Chunsuttitwat S., Vaughn D.W., Green S., Ennis F.A., Rothman A.L., Libraty D.H. (2004). Relationship of Preexisting Dengue Virus (DV) Neutralizing Antibody Levels to Viremia and Severity of Disease in a Prospective Cohort Study of DV Infection in Thailand. J. Infect. Dis..

[79] Wang T.T., Sewatanon J., Memoli M.J., Wrammert J., Bournazos S., Bhaumik S.K., Pinsky B.A., Chokephaibulkit K., Onlamoon N., Pattanapanyasat K. (2017). IgG Antibodies to Dengue Enhanced for FcgRIIIA Binding Determine Disease Severity Downloaded From. Science.

[80] Sastre D.E., Bournazos S., Du J., Boder E.J., Edgar J.E., Azzam T., Sultana N., Huliciak M., Flowers M., Yoza L. (2024). Potent Efficacy of an IgG-Specific Endoglycosidase against IgG-Mediated Pathologies. Cell.

[81] Mahan A.E., Jennewein M.F., Suscovich T., Dionne K., Tedesco J., Chung A.W., Streeck H., Pau M., Schuitemaker H., Francis D. (2016). Antigen-Specific Antibody Glycosylation Is Regulated via Vaccination. PLoS Pathog..

[82] Parekh R., Roitt I., Isenberg D., Dwek R., Rademacher T. (1988). Age-Related Galactosylation of the N-Linked Oligosaccharides of Human Serum IgG. J. Exp. Med..

[83] De Haan N., Reiding K.R., Driessen G., Van Der Burg M., Wuhrer M. (2016). Changes in Healthy Human IgG Fc-Glycosylation after Birth and during Early Childhood. J. Proteome Res..

[84] Sun W., Jian X., Zhang J., Meng X., Wang H., Zheng D., Wu L., Wang Y. (2024). The Causality between Human Immunoglobulin G (IgG) N-Glycosylation and Aging: A Mendelian Randomization Study. Molecules.

[85] Jennewein M.F., Kosikova M., Noelette F.J., Radvak P., Boudreau C.M., Campbell J.D., Chen W.H., Xie H., Alter G., Pasetti M.F. (2022). Functional and Structural Modifications of Influenza Antibodies during Pregnancy. iScience.

[86] Ruhaak L.R., Kim K., Stroble C., Taylor S.L., Hong Q., Miyamoto S., Lebrilla C.B., Leiserowitz G. (2016). Protein-Specific Differential Glycosylation of Immunoglobulins in Serum of Ovarian Cancer Patients. J. Proteome Res..

[87] Lomax-Browne H.J., Robertson C., Antonopoulos A., Leathem A.J.C., Haslam S.M., Dell A., Dwek M.V. (2019). Serum IgA1 Shows Increased Levels of A2,6-Linked Sialic Acid in Breast Cancer. Interface Focus.

[88] Zhang S., Cao X., Liu C., Li W., Zeng W., Li B., Chi H., Liu M., Qin X., Tang L. (2019). N-Glycopeptide Signatures of IgA2 in Serum from Patients with Hepatitis b Virus-Related Liver Diseases. Mol. Cell. Proteom..

[89] Wang J., Balog C.I.A., Stavenhagen K., Koeleman C.A.M., Scherer H.U., Selman M.H.J., Deelder A.M., Huizinga T.W.J., Toes R.E.M., Wuhrer M. (2011). Fc-Glycosylation of IgG1 Is Modulated by B-Cell Stimuli. Mol. Cell. Proteom..

[90] Cao Y., Song Z., Guo Z., Zhao X., Gong Y., Zhao K., Qu C., Huang Y., Li Y., Gao Y. (2022). Cytokines in the Immune Microenvironment Change the Glycosylation of IgG by Regulating Intracellular Glycosyltransferases. Front. Immunol..

[91] Lofano G., Gorman M.J., Yousif A.S., Yu W.-H., Fox J.M., Dugast A.-S., Ackerman M.E., Suscovich T.J., Weiner J., Barouch D. (2018). Antigen-Specific Antibody Fc Glycosylation Enhances Humoral Immunity via the Recruitment of Complement. Sci. Immunol..

[92] Van Coillie J., Pongracz T., Rahmöller J., Chen H.J., Geyer C.E., van Vught L.A., Buhre J.S., Šuštić T., van Osch T.L.J., Steenhuis M. (2023). The BNT162b2 MRNA SARS-CoV-2 Vaccine Induces Transient Afucosylated IgG1 in Naive but Not in Antigen-Experienced Vaccinees. eBioMedicine.

[93] Selman M.H.J., De Jong S.E., Soonawala D., Kroon F.P., Adegnika A.A., Deelder A.M., Hokke C.H., Yazdanbakhsh M., Wuhrer M. (2012). Changes in Antigen-Specific IgG1 Fc N-Glycosylation upon Influenza and Tetanus Vaccination. Mol. Cell. Proteom..

[94] Wang T.T., Maamary J., Tan G.S., Bournazos S., Davis C.W., Krammer F., Schlesinger S.J., Palese P., Ahmed R., Ravetch J.V. (2015). Anti-HA Glycoforms Drive B Cell Affinity Selection and Determine Influenza Vaccine Efficacy. Cell.

[95] Buhre J.S., Pongracz T., Künsting I., Lixenfeld A.S., Wang W., Nouta J., Lehrian S., Schmelter F., Lunding H.B., Dühring L. (2023). MRNA Vaccines against SARS-CoV-2 Induce Comparably Low Long-Term IgG Fc Galactosylation and Sialylation Levels but Increasing Long-Term IgG4 Responses Compared to an Adenovirus-Based Vaccine. Front. Immunol..

[96] Van Coillie J., Pongracz T., Šuštić T., Wang W., Nouta J., Le Gars M., Keijzer S., Linty F., Cristianawati O., Keijser J.B.D. (2023). Comparative Analysis of Spike-Specific IgG Fc Glycoprofiles Elicited by Adenoviral, MRNA, and Protein-Based SARS-CoV-2 Vaccines. iScience.

[97] Farkash I., Feferman T., Cohen-Saban N., Avraham Y., Morgenstern D., Mayuni G., Barth N., Lustig Y., Miller L., Shouval D.S. (2021). Anti-SARS-CoV-2 Antibodies Elicited by COVID-19 MRNA Vaccine Exhibit a Unique Glycosylation Pattern. Cell Rep..

[98] Bartsch Y.C., Eschweiler S., Leliavski A., Lunding H.B., Wagt S., Petry J., Lilienthal G.M., Rahmöller J., de Haan N., Hölscher A. (2020). IgG Fc Sialylation Is Regulated during the Germinal Center Reaction Following Immunization with Different Adjuvants. J. Allergy Clin. Immunol..

[99] Vaccari M., Gordon S.N., Fourati S., Schifanella L., Liyanage N.P.M., Cameron M., Keele B.F., Shen X., Tomaras G.D., Billings E. (2016). Adjuvant-Dependent Innate and Adaptive Immune Signatures of Risk of SIVmac251 Acquisition. Nat. Med..

[100] Liu J.K.H. (2014). The History of Monoclonal Antibody Development—Progress, Remaining Challenges and Future Innovations. Ann. Med. Surg..

[101] Crescioli S., Kaplon H., Wang L., Visweswaraiah J., Kapoor V., Reichert J.M. (2025). Antibodies to Watch in 2025. mAbs.

[102] Wang T., Liu L., Voglmeir J. (2022). MAbs N-Glycosylation: Implications for Biotechnology and Analytics. Carbohydr. Res..

[103] Monoclonal Antibody Therapeutics Market Growth, Drivers, and Opportunities. https://www.marketsandmarkets.com/Market-Reports/monoclonal-antibody-mabs-therapeutics-market-115323820.html.

[104] Thakkar S., Chopra A., Nagendra L., Kalra S., Bhattacharya S. (2023). Teplizumab in Type 1 Diabetes Mellitus: An Updated Review. touchREV. Endocrinol..

[105] Xu N., Ma C., Ou J., Sun W.W., Zhou L., Hu H., Liu X.M. (2017). Comparative Proteomic Analysis of Three Chinese Hamster Ovary (CHO) Host Cells. Biochem. Eng. J..

[106] Kunert R., Reinhart D. (2016). Advances in Recombinant Antibody Manufacturing. Appl. Microbiol. Biotechnol..

[107] William R.S., Lila M. (2012). Strohl Therapeutic Antibody Classes. Therapeutic Antibody Engineering.

[108] Gangwar N., Dixit N., Rathore A.S. (2025). N-Glycosylation Modulators for Targeted Manipulation of Glycosylation for Monoclonal Antibodies. Appl. Microbiol. Biotechnol..

[109] Beck A., Liu H. (2019). Macro-and Micro-Heterogeneity of Natural and Recombinant IgG Antibodies. Antibodies.

[110] Liu L. (2018). Pharmacokinetics of Monoclonal Antibodies and Fc-Fusion Proteins. Protein Cell.

[111] Singh S.K., Lee K.H. (2022). Characterization of Monoclonal Antibody Glycan Heterogeneity Using Hydrophilic Interaction Liquid Chromatography-Mass Spectrometry. Front. Bioeng. Biotechnol..

[112] Anthony R.M., Nimmerjahn F., Ashline D.J., Reinhold V.N., Paulson J.C., Ravetch J.V. (2008). A Recombinant IgG Fc that Recapitulates the Anti-Inflammatory Activity of IVIG.

[113] Siebert H.C., Rosen J., Seyrek K., Kaltner H., André S., Bovin N.V., Nyholm P.G., Sinowatz F., Gabius H.J. (2006). A2,3/A2,6-Sialylation of N-Glycans: Non-Synonymous Signals with Marked Developmental Regulation in Bovine Reproductive Tracts. Biochimie.

[114] Chung C.Y., Wang Q., Yang S., Yin B., Zhang H., Betenbaugh M. (2017). Integrated Genome and Protein Editing Swaps α-2,6 Sialylation for α-2,3 Sialic Acid on Recombinant Antibodies from CHO. Biotechnol. J..

[115] Lin N., Mascarenhas J., Sealover N.R., George H.J., Brooks J., Kayser K.J., Gau B., Yasa I., Azadi P., Archer-Hartmann S. (2015). Chinese Hamster Ovary (CHO) Host Cell Engineering to Increase Sialylation of Recombinant Therapeutic Proteins by Modulating Sialyltransferase Expression. Biotechnol. Prog..

[116] Tuntland T., Ethell B., Kosaka T., Blasco F., Zang R., Jain M., Gould T., Hoffmaster K. (2014). Implementation of Pharmacokinetic and Pharmacodynamic Strategies in Early Research Phases of Drug Discovery and Development at Novartis Institute of Biomedical Research. Front. Pharmacol..

[117] Lodge J., Kajtar L., Duxbury R., Hall D., Burley G.A., Cordy J., Yates J.W.T., Rattray Z. (2025). Quantifying Antibody Binding: Techniques and Therapeutic Implications. mAbs.

[118] Reusch J., Andersen J.T., Rant U., Schlothauer T. (2024). Insight into the Avidity–Affinity Relationship of the Bivalent, PH-Dependent Interaction between IgG and FcRn. mAbs.

[119] Betts A., Keunecke A., van Steeg T.J., van der Graaf P.H., Avery L.B., Jones H., Berkhout J. (2018). Linear Pharmacokinetic Parameters for Monoclonal Antibodies Are Similar within a Species and across Different Pharmacological Targets: A Comparison between Human, Cynomolgus Monkey and HFcRn Tg32 Transgenic Mouse Using a Population-Modeling Approach. mAbs.

[120] Zhu W., Zhou Y., Guo L., Feng S. (2024). Biological Function of Sialic Acid and Sialylation in Human Health and Disease. Cell Death Discov..

[121] Boune S., Hu P., Epstein A.L., Khawli L.A. (2020). Principles of N-linked Glycosylation Variations of Igg-based Therapeutics: Pharmacokinetic and Functional Considerations. Antibodies.

[122] Keizer R.J., Huitema A.D.R., Schellens J.H.M., Beijnen J.H. (2010). Clinical Pharmacokinetics of Therapeutic Monoclonal Antibodies. Clin. Pharmacokinet..

[123] Datta-Mannan A. (2019). Mechanisms Influencing the Pharmacokinetics and Disposition of Monoclonal Antibodies and Peptides. Drug Metab. Dispos..

[124] Glassman P.M., Abuqayyas L., Balthasar J.P. (2015). Assessments of Antibody Biodistribution. J. Clin. Pharmacol..

[125] Wolf B., Piksa M., Beley I., Patoux A., Besson T., Cordier V., Voedisch B., Schindler P., Stöllner D., Perrot L. (2022). Therapeutic Antibody Glycosylation Impacts Antigen Recognition and Immunogenicity. Immunology.

[126] Matucci A., Nencini F., Vivarelli E., Bormioli S., Maggi E., Vultaggio A. (2021). Immunogenicity-Unwanted Immune Responses to Biological Drugs–Can We Predict Them?. Expert. Rev. Clin. Pharmacol..

[127] Mastrangeli R., Audino M.C., Palinsky W., Broly H., Bierau H. (2020). The Formidable Challenge of Controlling High Mannose-Type N-Glycans in Therapeutic MAbs. Trends Biotechnol..

